# Knowledge, Attitudes, and Perceptions Towards Human Papillomavirus (HPV) Vaccination Among Adult Women in Primary Health Care Centers in Makkah, Saudi Arabia

**DOI:** 10.7759/cureus.44157

**Published:** 2023-08-26

**Authors:** Yousef M Turki, Jehad Alqurashi

**Affiliations:** 1 Preventive Medicine, Preventive Medicine Board Program, Makkah, SAU; 2 Public Health, Ministry of Health, Saudi Board of Preventive Medicine, Mecca, SAU

**Keywords:** womens health, human papillomavirus (hpv), human papilloma virus vaccine, human papilloma virus vaccination, health education, public awareness, vaccine uptake, cervical cancer, hpv infection

## Abstract

Background: Human papillomavirus (HPV) is linked to cervical cancer, which is prevalent in Saudi Arabia. While HPV vaccines are effective, their coverage remains low in low- and middle-income countries. Public awareness of HPV vaccination is also limited. The study examines public awareness of cervical cancer, HPV, the HPV vaccine, and factors that hinder vaccine uptake.

Objectives: To assess the levels of knowledge, attitudes, and perceptions towards HPV vaccination among women aged 16 years and above in Makkah, Saudi Arabia.

Methods: An analytical cross-sectional study was conducted using an interview questionnaire. A stratified sampling technique was used to select a representative sample of 534 female patients aged 16 years and older who visited primary healthcare centers in Makkah. The interview questionnaire included questions related to sociodemographic characteristics, knowledge about HPV vaccination, attitudes and perceptions toward HPV vaccination, and sources of information about HPV infection and vaccines. The data were analyzed using descriptive statistics and chi-square tests.

Results: The majority of participants were aged between 21 and 40 years (76.4%), Saudi (90.3%), and had a higher education level (73.4%). Only a small proportion (1.9%-39%) of participants correctly answered most of the questions related to HPV vaccination, while a few questions were answered correctly by a larger proportion (41.6%-59.6%), highlighting the need for educational programs to increase awareness about the HPV vaccine. The internet and social media were the most prevalent sources of information about HPV infection and vaccines (48.4%). A majority of participants expressed willingness to receive the HPV vaccine if offered by the healthcare sector at no cost (65.5%). Concerns included fear of injection (27.7%), cost (23.2%), and potential refusal from family or community (9.7%). A significant proportion believed that there is a need for educational sessions to increase awareness about the HPV vaccine in their community (82.8%) and that increased knowledge about HPV vaccines would lead to greater acceptability (83.9%). Age, nationality, marital status, number of children, educational status, occupation, and average monthly income were significantly associated with knowledge about HPV vaccination (p < 0.05). Age and educational status were significantly associated with attitudes towards HPV vaccination (p < 0.05).

Conclusion: The lack of knowledge about HPV vaccination among adult women in Makkah, Saudi Arabia, is concerning, as it may result in low vaccine uptake rates and an increased incidence of HPV-related diseases. Therefore, targeted educational programs and awareness campaigns are crucial to enhance knowledge and promote the uptake of the HPV vaccine. These programs should be designed to provide accurate information about the prevalence of HPV, its associated risks, and the benefits of vaccination.

## Introduction

The human papillomavirus (HPV) is a small virus that contains a double-stranded, non-enveloped (DNA) genome [1]. Infections with HPV primarily lead to the development of cervical cancer. According to the World Health Organization (WHO), cervical cancer accounts for 84% of all HPV-associated cancer cases [2]. Given its associated morbidity and mortality rates, as well as its global prevalence, cervical cancer poses a significant burden on global health [2]. Furthermore, HPV infections are primarily transmitted through sexual contact and affect 14 million people annually [3].

The global prevalence of cervical cancer is very high, with over 500,000 cases recorded annually and around 250,000 women dying from the disease [4]. In the Middle Eastern and North African (MENA) region, the prevalence of HPV in the cervical cancer population is 81% [5]. In Saudi Arabia, data resources suggest that more than 350 women are diagnosed with cervical cancer each year, with over 170 dying from this deadly disease [6]. Women in their reproductive age or sexually active stage, particularly adolescent women, are at increased risk of contracting HPV infection and developing cervical cancer [7]. Human papillomavirus infections pose a particular threat to the female population, as cervical cancer is the second most common cancer in women worldwide [7].

The human papillomavirus vaccines effectively reduce the risk of cervical cancer [8]. In a population-based follow-up study, it was reported that women who had previously received the HPV vaccine were at a lower risk of developing invasive cervical cancer compared to those who had not been vaccinated [8]. According to data published by the WHO, HPV vaccination has been included in the national vaccination programs of 117 countries worldwide [9]. However, the coverage of HPV vaccination is still lower in low- and middle-income countries compared to high-income countries [9]. Additionally, the United Nations Children's Fund (UNICEF) data shows a lack of information regarding HPV vaccine coverage in the majority of Arab countries, including Saudi Arabia [10].

The high incidence rate of cervical cancer and HPV infections among women is alarming, despite the availability of efficient vaccines to control HPV infection. Although the utilization rate of the HPV vaccine is average, the comparative uptake rate is lower than other vaccines [11]. Even in developed countries like the United States (US), public awareness about HPV vaccination has been reported to be limited [11,12]. In Saudi Arabia, data related to HPV vaccine acceptance and coverage are not available, and studies regarding HPV awareness are limited [9]. Therefore, it is crucial to conduct further studies to analyze the current level of public awareness regarding cervical cancer, HPV, the HPV vaccine, and factors that hinder vaccine uptake. The objective of this cross-sectional research is to study the knowledge of this subject.

Hence, it is important to undertake additional investigations aimed at analyzing the existing extent of public awareness regarding cervical cancer, HPV, and HPV vaccination. The objective of this cross-sectional study is to assess the levels of knowledge, attitudes, and perceptions toward HPV vaccination among women aged 16 years and older in Makkah, Saudi Arabia.

## Materials and methods

Study design

An analytical cross-sectional study was conducted using an interview questionnaire to explore the knowledge, attitudes, and perceptions of adult women regarding HPV vaccination, in Makkah, Kingdom of Saudi Arabia. The study was carried out between April 2023 and January 2023.

Sampling and participants

All women aged 16 years and older, residing in Makkah, and visiting a primary health care center (PHCC) were eligible to participate in the study. There were no specific exclusion criteria applied other than the inability to obtain informed consent for participation. The sample size was calculated to detect an unknown prevalence (P = 50%) of the population with adequate knowledge due to the fact that no previous studies have been conducted in Makkah with ±0.05 precision and a 95% confidence level. The calculation used the formula was: (n = (Z2 * P (1 - P))/e2). The final calculated sample size was 384 women.

One PHCC from each region of Makkah (east, west, north, and south) was randomly selected from a total of three PHCCs in each region. The random selection was done using random number generator software. The Security Forces Primary Health Care Center was included from the east, the Alzaidi Primary Health Care Center from the west, the Altan'eem Primary Health Care Center from the north, and the Alkakiyyah Primary Health Care Center from the south. Participants were recruited from the selected PHCCs during the weekdays using systematic random sampling (every fifth patient). The first patient was assigned using a simple random number generator. The sample was considered satisfied when each selected PHCC recorded at least 100 patients. The sample size was open to higher numbers to increase the study power.

Data collection

The interview questionnaire was developed based on a modified version of a validated questionnaire used in previous studies [13,14]. We also explored other studies to include a wide range of variables that may influence vaccine perception [15,16]. The questionnaire included questions related to sociodemographic characteristics, knowledge, attitudes, perceptions, and sources of information about HPV infection and vaccines. The interview-validated questionnaire was administered after obtaining informed consent. A participant who correctly answered 75% or more of the questions (27 points out of 36) was considered to have good knowledge and practice about HPV vaccines.

Data analysis

Data were coded, entered, and analyzed using IBM SPSS software version 23 (IBM Corp., Armonk, NY, USA). The data were analyzed using descriptive statistics, including frequencies and percentages. A chi-square (χ²) test was used to examine the associations between categorical variables, and Fisher exact tests were used for tables with > 20% of expected cell counts less than five. The items of each sub-scale (knowledge, attitude, and perception) were computed to calculate the total scores for each domain, giving one point for each correct answer. The total scores were then categorized according to the median and interquartile range (IQR) to give a comparable sample size for all categories. In terms of knowledge questions, scores falling below 25% were classified as poor, while scores ranging from 25% to 50% were categorized as moderate. A good level of knowledge was assigned to scores surpassing 50%. Similarly, for attitude and perception, categories were determined using the median; scores exceeding 50% of the total were considered positive. A p-value of less than 0.05 was considered statistically significant.

Ethical considerations

This study was conducted in accordance with the ethical principles outlined in the Declaration of Helsinki. The research protocol was approved by the Institutional Review Board (IRB) at the University of Makkah, Makkah, Saudi Arabia (IRB approval number: SFHM-HM-FRM-018). Prior to starting the interview, written informed consent was obtained from all participants. The participants were informed about the purpose of the study, their rights as research participants, and the confidentiality of their data. The interviews were conducted in a private setting to ensure the privacy and confidentiality of the participants. The information collected from the participants was used solely for research purposes and was not disclosed to any third parties. The study did not pose any physical or psychological risks to the participants.

## Results

Sociodemographic characteristics of participants assessed for knowledge, attitude, and perception toward the HPV vaccine

A total of 534 participants were included in the study, all of whom were female. Table 1 displays the sociodemographic characteristics of female participants in Makkah, who were assessed for their knowledge, attitude, and perception towards the HPV vaccine.

**Table 1:** The sociodemographic characteristics of participants assessed for knowledge, attitude, and perception towards the HPV vaccine SAR: Saudi Riyal

Variable		Frequency (n=534)	Percentage (%)
Gender	Female	534	100.0
Age	< 20 Years	40	7.5
	21-40 Years	408	76.4
	41-60 Years	78	14.6
	>60 Years	8	1.5
Nationality of the women living in Makkah	Non-Saudi	52	9.7
	Saudi	482	90.3
Occupation	Student	112	21.0
	Employed	170	31.8
	Unemployed	234	43.8
	Other	18	3.4
Marital status	Single	220	41.2
	Married	274	51.3
	Divorced	22	4.1
	Widow	18	3.4
No. of children	0	238	44.6
	1	44	8.2
	2	64	12.0
	3	62	11.6
	4 or more	126	23.6
Educational status	Uneducated	6	1.1
	Primary	4	.7
	Middle-school	8	1.5
	Secondary	124	23.2
	Higher education (Bachelor's degree and above)	392	73.4
Average monthly income	< 999 SAR	182	34.1
	1000-3999 SAR	164	30.7
	4000-7999 SAR	56	10.5
	8000-9999 SAR	46	8.6
	>10000 SAR	86	16.1

In terms of age distribution, the majority of participants (76.4%) were aged between 21 and 40 years. Regarding nationality, the vast majority of women (90.3%) were Saudi, while 9.8% were non-Saudi but residing in Makkah. The occupation distribution of participants revealed that a significant proportion was unemployed (43.8%), followed by those who were employed (31.8%) and students (21.0%). In terms of marital status, the majority of participants were married (51.3%). The largest group consisted of participants with no children (44.6%). In terms of educational status, the majority of participants had a higher education level (73.4%), including bachelor's degrees and above. Regarding average monthly income, the highest percentage of participants fell into the income range of <999 Saudi Riyals (34.1%).

Questions with correct answers assessing the knowledge of adult women HPV vaccination

**Figure 1:**
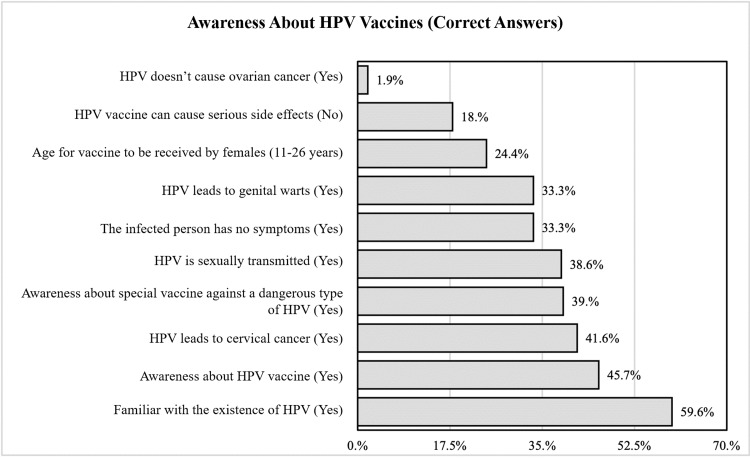
Questions with correct answers to assess the knowledge of adult women in Makkah about HPV vaccination HPV: human papillomavirus

The questions used to assess the knowledge of adult women in Makkah regarding HPV vaccination and the percentages of participants who answered each question correctly are shown in Figure 1.

Only a small proportion of participants (1.9%) correctly answered that HPV does not cause ovarian cancer. Similarly, a relatively low percentage (18%) knew that the HPV vaccine does not cause serious side effects. A large proportion of participants (41.6%) correctly recognized that HPV can lead to cervical cancer. Awareness of the HPV vaccine was higher, with 45.7% of the participants indicating knowledge about it. The majority of participants (59.6%) stated that they were familiar with the existence of HPV.

**Figure 2:**
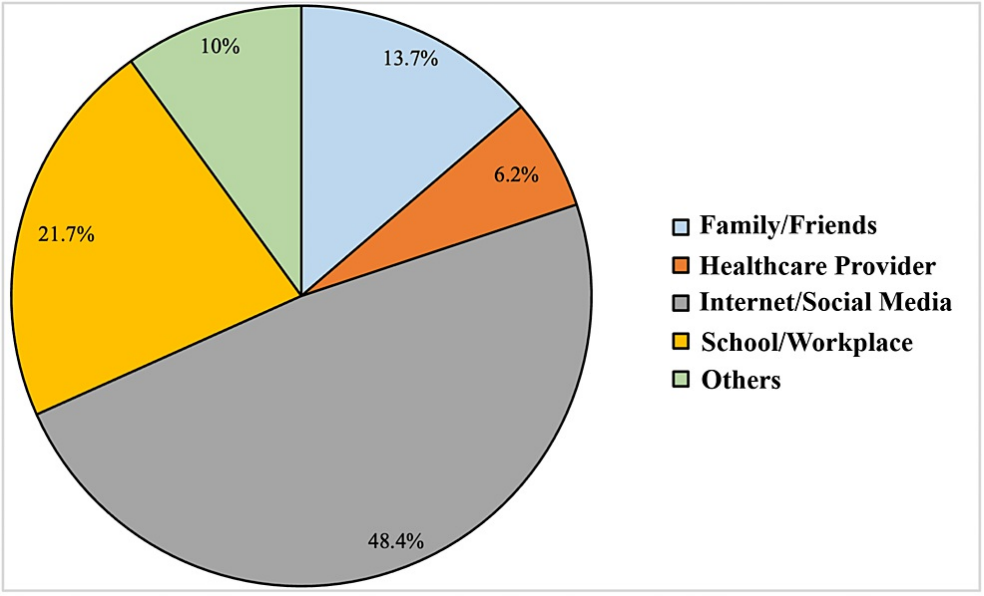
Sources of information about human papillomavirus (HPV) infection and vaccines

Figure 2 shows the source of information about HPV infection and vaccines.

The Internet and social media are the most prevalent sources of information about HPV infection and vaccines for women, with a proportion of 48.4%. Other sources are shown in Figure 2.

Questions assessing the attitude and perceptions of adult Saudi women towards HPV infection and vaccination

Table 2 provides an overview of the attitudes and perceptions of adult Saudi women towards HPV infection and vaccination.

**Table 2:** Questions assessing the attitude and perceptions of adult Saudi women toward HPV infection and vaccination HPV: human papillomavirus

Statement	Frequency (n=534)	Percent
Attitude towards HPV infection and vaccines		
Have you ever been tested/screened for HPV (Yes)	32	6.0
Will you receive the HPV vaccine if offered by the healthcare sector without any cost? (Yes)	350	65.5
Will you receive the HPV vaccine if offered by the healthcare sector at some cost? (Yes)	200	37.5
Will you vaccinate your daughter with the HPV vaccine without any cost? (Yes)	322	60.3
Will you vaccinate your daughter with the HPV vaccine at some cost? (Yes)	228	42.7
Would you hesitate to get the HPV vaccine due to fear of injections? (Yes)	148	27.7
Would you hesitate to get the HPV vaccine due to cost? (Yes)	124	23.2
Would you hesitate to get the HPV vaccine due to refusal from family/community? (Yes)	52	9.7
Would you like to recommend others to be vaccinated with the HPV vaccine? (Yes)	290	54.3
Perception of HPV infection and vaccines? (Yes)		
Do you think you are at risk of HPV infection? (Yes)	106	19.9
Do you have any questions related to the HPV vaccines (availability, safety, efficiency) in your mind? (Yes)	248	46.4
Do you think there is a need for educational sessions to increase awareness about the HPV vaccine in your community? (Yes)	442	82.8
Do you think there will be more acceptability towards the HPV vaccine if people get to know more about HPV vaccines? (Yes)	448	83.9

Among the participants, only 6.0% reported having been tested or screened for HPV. A majority (65.5%) expressed willingness to receive the HPV vaccine if offered by the healthcare sector at no cost, while 37.5% would consider it at some cost. A significant proportion (82.8%) believed that there is a need for educational sessions to increase awareness about the HPV vaccine in their community, and 83.9% felt that increased knowledge about HPV vaccines would lead to greater acceptability.

Association of different demographic features with the level of knowledge of women about HPV vaccination

Table 3 displays the associations between different demographic features and the level of knowledge about HPV vaccination among participants.

**Table 3:** Association of different demographic features with the level of knowledge of women about HPV vaccination HPV: human papillomavirus; SAR: Saudi Riyal

Variable		Knowledge about HPV vaccine			p-value
		Poor knowledge	Moderate knowledge	High knowledge	
Age	< 20 years	18 (11.7)	14 (7.9)	8 (4)	0.002*
	21-40 years	108 (70.1)	132 (74.2)	168 (83.2)	
	41-60 years	22 (14.3)	32 (18)	24 (11.9)	
	>60 years	6 (3.9)	0 (0)	2 (1)	
Nationality	Non-Saudi	24 (15.6)	12 (6.7)	16 (7.9)	0.014*
	Saudi	130 (84.4)	166 (93.3)	186 (92.1)	
Marital status	Single	72 (49.3)	74 (41.6)	70 (34.7)	0.029*
	Married	62 (40.3)	92 (51.7)	120 (59.4)	
	Divorced	10 (6.5)	6 (3.4)	6 (3)	
	Widow	6 (3.9)	6 (3.4)	6 (3)	
No. of children	0	80 (51.9)	82 (46.1)	76 (37.6)	<0.001*
	1	6 (3.9)	12 (6.7)	26 (12.9)	
	2	18 (11.7)	8 (4.5)	38 (18.8)	
	3	28 (18.2)	16 (9)	18 (8.9)	
	4 or more	22 (14.3)	60 (33.7)	44 (21.8)	
Educational status	Uneducated	6 (3.9)	0 (0)	0 (0)	<0.001*
	Primary	4 (2.6)	0 (0)	0 (0)	
	Middle school	2 (1.3)	4 (2.2)	2 (1)	
	Secondary	38 (24.7)	54 (30.3)	32 (15.8)	
	Higher	104 (67.5)	120 (67.4)	168 (83.2)	
Occupation	Student	38 (24.7)	42 (23.6)	32 (15.8)	<0.001*
	Employed	38 (24.7)	38 (1.3)	94 (46.5)	
	Unemployed	74 (48.1)	94 (52.8)	66 (32.7)	
	Other	4 (2.6)	4 (2.2)	10 (5)	
Average monthly income	< 999 SAR	74 (48.1)	66 (37.1)	42 (20.8)	<0.001*
	1000-3999 SAR	36 (23.4)	64 (36)	64 (31.7)	
	4000-7999 SAR	22 (14.3)	16 (9)	18 (8.9)	
	8000-9999 SAR	12 (7.8)	12 (6.7)	22 (10.9)	
	>10000 SAR	10 (6.5)	20 (11.2)	56 (27.7)	

The findings provide insights into how these demographic factors relate to knowledge levels. Participants in the age group 21-40 years (83.2%) showed a significant association with a high level of knowledge about HPV vaccination (p = 0.002*). Marital status demonstrated a significant association with knowledge, with married individuals (59.4%) having a higher level of knowledge about HPV (p = 0.029*). The number of children also showed a significant association with knowledge about HPV vaccination. Participants with no children (37.4%) tended to have the highest knowledge levels, followed by those with four or more children, who showed a higher level of knowledge (p = 0.001*). Educational status exhibited a significant association with knowledge; participants with higher educational attainment had higher knowledge levels, with the highest level observed among those with higher education (83.2%) (p = 0.001*). Occupation showed a significant association with knowledge. Participants who were employed had the highest level of knowledge, while students had the lowest (p = 0.001*). Average monthly income also demonstrated a significant association with knowledge. Participants with higher incomes tended to have higher knowledge levels, with the highest knowledge level observed among those with an income above 1000-3999 Saudi Riyals (p < 0.001).

Association of different demographic features with positive or negative attitudes of women towards the HPV vaccination

Table 4 displays the associations between different demographic features and women's attitudes, positive or negative, towards HPV vaccination.

**Table 4:** Association of different demographic features with positive or negative attitudes of women towards HPV vaccination HPV: human papillomavirus; SAR: Saudi Riyal

Variable		Attitude towards HPV vaccine		P-value
		Negative attitude	Positive attitude	
Age	< 20 years	6 (6.4)	34 (7.7)	<0.001*
	21-40 years	72 (76.6)	336 (76.6)	
	41-60 years	10 (10.6)	68 (15.5)	
	>60 years	6 (6.4)	2 (0.5)	
Nationality	Non-Saudi	10 (10.6)	42 (9.5)	0.746
	Saudi	84 (89.4)	398 (90.5)	
Marital status	Single	30 (31.9)	190 (43.2)	0.179
	Married	54 (57.4)	220 (50)	
	Divorced	6 (6.4)	16 (3.6)	
	Widow	4 (4.3)	14 (3.2)	
No. of children	0	36 (38.3)	202 (45.9)	0.068
	1	8 (8.5)	36 (8.2)	
	2	6 (6.4)	58 (13.2)	
	3	16 (17)	46 (10.5)	
	4 or more	28 (29.8)	98 (22.3)	
Educational Status	Uneducated	4 (4.3)	2 (0.5)	0.003*
	Primary	2 (2.1)	2 (0.5)	
	Middle school	0 (0)	8 (1.8)	
	Secondary	26 (27.7)	98 (22.3)	
	Higher	62 (66)	330 (75)	
Occupation	Student	22 (23.4)	90 (20.5)	0.114
	Employed	20 (21.3)	150 (34.1)	
	Unemployed	48 (51.1)	186 (42.3)	
	Other	4 (4.3)	14 (3.2)	
Average monthly income	< 999 SAR	32 (34.0)	150 (34.1)	0.064
	1000-3999 SAR	34 (36.2)	130 (29.5)	
	4000-7999 SAR	12 (12.8)	44 (10.0)	
	8000-9999 SAR	10 (10.6)	36 (8.2)	
	>10000 SAR	6 (6.4)	80 (18.2)	

Age exhibited a significant association with attitudes towards HPV vaccination. Participants aged 21-40 had a higher proportion of positive attitudes towards HPV vaccination compared to negative attitudes (p = 0.001). The educational status also demonstrated a significant association with attitudes. Participants with higher levels of education tended to have more positive attitudes towards HPV vaccination (p = 0.003). Nationality, marital status, number of children, occupation, and average monthly income did not exhibit significant associations with attitudes toward HPV vaccination (p > 0.05).

Association of different demographic features with positive or negative perceptions of women towards HPV vaccination

Table 5 shows the associations between different demographic features and the positive or negative perceptions of women toward HPV vaccination.

**Table 5:** Association of different demographic features with positive or negative perceptions of women towards HPV vaccination HPV: human papillomavirus; SAR: Saudi Riyal

Variable		Perception of HPV vaccine		p-value
		Negative perception	Positive perception	
Age	< 20 years	4 (7.1)	36 (7.5)	0.216
	21-40 years	46 (82.1)	363 (75.5)	
	41-60 years	4 (7.1)	74 (15.5)	
	>60 years	2 (3.6)	6 (1.3)	
Nationality	Non-Saudi	4 (7.1)	48 (10.0)	0.489
	Saudi	52 (92.9)	430 (90.0)	
Marital status	Single	20 (35.7)	200 (41.8)	0.819
	Married	32 (57.1)	242 (50.6)	
	Divorced	2 (3.6)	20 (4.2)	
	Widow	2 (3.6)	16 (3.3)	
No. of children	0	20 (35.7)	218 (45.6)	0.008*
	1	10 (17.9)	34 (7.1)	
	2	2 (3.6)	62 (13.0)	
	3	6 (10.7)	56 (11.7)	
	4 or more	18 (32.1)	108 (22.6)	
Educational status	Uneducated	2 (3.6)	4 (0.8)	0.302
	Primary	0 (0)	4 (0.8)	
	Middle school	0 (0)	8 (1.7)	
	Secondary	14 (25)	110 (23.0)	
	Higher	40 (71.4)	352 (73.6)	
Occupation	Student	10 (17.9)	102 (21.3)	0.006*
	Employed	8 (14.3)	162 (33.9)	
	Unemployed	36 (63.3)	198 (41.4)	
	Other	2 (3.6)	16 (3.3)	
Average monthly income	< 999 SAR	20 (35.7)	162 (33.9)	0.005*
	1000-3999 SAR	22 (39.3)	142 (29.7)	
	4000-7999 SAR	10 (17.9)	46 (9.6)	
	8000-9999 SAR	4 (7.1)	42 (8.8)	
	>10000 SAR	0 (0)	86 (18)	

The results indicate the significance level (Sig.) for each association. The number of children demonstrated a significant association with perception. Participants with no children had a positive perception toward HPV vaccination (p = 0.008). Similarly, occupation showed a significant association with perception. Unemployed participants (41.4%) had more positive perceptions toward HPV vaccination (p = 0.006). Finally, income showed a significant association with perception. Participants with an income less than 999 SAR (33.9%) had more positive perceptions toward HPV vaccination (p = 0.005)*. Age, nationality, marital status, and educational status did not show significant associations with perceptions of HPV vaccination (p > 0.05).

## Discussion

This study assessed the knowledge, attitudes, and perceptions of adult women in Makkah, Saudi Arabia, regarding the HPV vaccine. The results showed limited knowledge of the virus but a positive attitude towards receiving the vaccine if offered for free. The study highlights the need for targeted educational interventions to promote positive attitudes and perceptions toward the vaccine and increase vaccine coverage.

In terms of sociodemographic characteristics, the majority of participants were between the ages of 21 and 40, indicating a relatively young sample. This age group is particularly important for HPV vaccination, as it falls within the recommended age range for vaccination (11-26 years old) [17]. Moreover, the study sample mainly consisted of Saudi women, emphasizing the necessity of targeting the local population for HPV education and vaccination efforts. Knowledge about HPV and its associated risks is essential for informed decision-making and preventive measures. In our study, only a small proportion of participants (1.9%) correctly answered that HPV does not cause ovarian cancer. This finding underscores a knowledge gap that targeted education and awareness campaigns need to address. It is consistent with previous studies that have reported low awareness of the relationship between HPV and ovarian cancer [18]. A relatively low percentage of participants (18%) knew that the HPV vaccine does not cause serious side effects. This finding is consistent with prior research indicating misconceptions and concerns about vaccine safety [19]. It underscores the importance of providing accurate information about vaccine safety to alleviate fears and increase vaccine acceptance. Regarding awareness of the consequences of HPV infection, a considerable proportion of participants (41.6%) recognized that HPV can lead to cervical cancer, and 33.3% recognized that it can cause genital warts. These findings are in line with previous studies that have shown varying levels of awareness regarding the link between HPV and cervical cancer [20,21]. However, it is important to note that a significant proportion of participants were still unaware of these risks, highlighting the need for comprehensive educational campaigns to improve knowledge.

In terms of the age range for females to receive the HPV vaccine, approximately one-fourth of participants (24.4%) correctly identified the range as 11-26 years old. This finding aligns with other studies that report suboptimal knowledge of the recommended age range for HPV vaccination [22]. It emphasizes the need for targeted educational interventions to ensure that women are aware of the optimal age for vaccination. The internet and social media were the primary sources of information about HPV infection and vaccines, accounting for 48.4% of responses. This trend reflects the growing influence of online platforms in distributing health information [22,23]. However, it also underscores the risk of misinformation and the need to ensure that accurate and dependable information is easily accessible to the public.

Regarding vaccine acceptance, the majority of participants (65.5%) expressed a willingness to receive the HPV vaccine if offered by the healthcare sector at no cost. This finding is encouraging and suggests a positive attitude towards vaccination. However, it is crucial to address concerns such as fear of injection (27.7%) and cost (23.2%), which were identified as barriers to vaccine uptake. Similar concerns have been reported in previous studies, emphasizing the need for targeted interventions to address these specific barriers [24]. Participants expressed a strong belief (82.8%) in the need for educational sessions to increase awareness about the HPV vaccine in their community. This finding highlights the perceived value of community-based educational initiatives, which have been shown to improve knowledge and vaccine acceptance [25,26]. It underscores the importance of implementing educational programs to bridge the knowledge gap and enhance community acceptance of HPV vaccination.

It is important to acknowledge that this study had some limitations. Firstly, the sample consisted solely of female participants from a specific region in Saudi Arabia, which may limit the generalizability of the findings to other populations. Additionally, the use of self-reported data may have introduced bias, and the cross-sectional design of the study prevents the establishment of causality. Future research could address these limitations by exploring larger and more diverse samples, employing longitudinal designs, and incorporating qualitative methods to gain a deeper understanding of the factors influencing knowledge, attitudes, and perceptions toward HPV vaccination. However, the study is notable for its utilization of a structured questionnaire to collect data, which allows for standardized data collection and analysis. Furthermore, the study employed a relatively large sample size that represented adult women in primary healthcare centers in Makkah, Saudi Arabia. Finally, the study provides valuable insights into the knowledge, attitudes, and perceptions of adult women regarding HPV vaccination in a region where research in this area is limited.

## Conclusions

The study shows that while there is limited knowledge about HPV and its associated risks, there is a positive attitude towards receiving the vaccine if offered for free. The internet and social media were the primary sources of information, and concerns such as fear of injection and cost were identified as barriers to vaccine uptake. However, participants expressed a strong belief in the need for educational sessions to increase awareness about the HPV vaccine in their community. The findings underscore the importance of targeted educational interventions to improve knowledge, address misconceptions, and promote positive attitudes and perceptions toward the vaccine. By addressing the identified knowledge gaps and overcoming barriers, healthcare providers and public health authorities can contribute to increasing HPV vaccine coverage and ultimately reduce the burden of HPV-related diseases in the population.
